# Thiol-disulfide homeostasis: an integrated approach with biochemical and clinical aspects

**DOI:** 10.3906/sag-2003-64

**Published:** 2020-11-03

**Authors:** Özcan EREL, Serpil ERDOĞAN

**Affiliations:** 1 Department of Medical Biochemistry, Faculty of Medicine, Ankara Yıldırım Beyazıt Universty, Ankara Turkey; 2 Department of Medical Biochemistry, Ankara City Hospital, Ankara Turkey

**Keywords:** Thiol, disulfide, oxidant stress, thiol-disulfide homeostasis

## Abstract

Dynamic thiol-disulfide homeostasis (TDH) is a new area has begun to attract more scrutiny. Dynamic TDH is reversal of thiol oxidation in proteins and represents the status of thiols (-SH) and disulfides (-S-S-). Organic compounds containing the sulfhydryl group is called thiol, composed of sulfur and hydrogen atoms. Disulfides are the most important class of dynamic, redox responsive covalent bonds build in between two thiol groups. For many years, thiol levels were analyzed by several methods. During last years, measurements of disulfide levels have been analyzed by a novel automated method, developed by Erel and Neselioglu. In this method, addition to thiol (termed as native thiol) levels, disulfide levels were also measured and sum of native thiol and disulfide levels were termed as total thiol. Therefore, TDH was begun to be understood in organism. In healthy humans, TDH is maintained within a certain range. Dysregulated dynamic TDH has been implicated several disorders with unknown etiology. A growing body of evidence has demonstrated that the thiol-disulfide homeostasis is involved in variety diseases, such as diabetes mellitus, hypertension, nonsmall cell lung cancer, familial Mediterranean fever (FMF), inflammatory bowel diseases, occupational diseases, gestational diabetes mellitus and preeclampsia. These results may elucidate some pathogenic mechanism or may be a predictor indicating diagnostic clue, prognostic marker or therapeutic sign. In conclusion, protection of the thiol-disulfide homeostasis is of great importance for the human being. Evidence achieved so far has proposed that thiol-disulfide homeostasis is an important issue needs to elucidate wholly.

## 1. Introduction 

Human life is maintained by oxygen and aerobic processes, and reactive oxygen species (ROS) are harmful by-products of human organism metabolism [1]. Reactive oxygen species, composed of several molecules such as superoxide anion radicals (O2-), hydrogen peroxide (H2O2), and hydroxyl radicals (-OH), are normally produced by cells during metabolic processes such as protein synthesis and mitochondrial metabolism [2].

The oxidative effects of ROS are neutralized by the antioxidant capacity of cells and this battle with oxidant stress maintains homeostasis [1]. Within the cell, the redox couples are controlled in a location-specific manner, especially in mitochondria, endoplasmic reticulum (ER) and nuclei [3]. Additionally, extracellular compartments supply defensive barriers against external oxidants. Cysteine (Cys) and its disulfide, cystine (CySS) compose the major low-molecular weight thiol/disulfide couple in human plasma. The Cys/CySS pool is central redox control point in the biological signaling [4,5].

## 2. Dynamic thiol-disulfide homeostasis

Organic compounds containing the sulfhydryl group is called thiol (-SH), composed of sulfur and hydrogen atoms. The thiols have high vulnerability to the oxidation due to their –SH group. Disulfides (-S-S-) are the most important class of dynamic, redox responsive covalent bonds build in between two thiol groups. Dynamic thiol-disulfide homeostasis (TDH) is reversal of thiol oxidation in proteins and represents the levels of thiols and disulfides. It is an important parameter associated with several biochemical processes, including regulation of protein function, stabilization of protein structure, protection of proteins against irreversible oxidation of cysteine residues, chaperon function, regulation of enzyme functions and transcription [6–9].

### 2.1. Thiol and disulfide detection

Up to date thiol, disulfide, sulfur-containing amino acid, reduced glutathione and oxidized glutathione levels were analyzed by several methods. 

Ellman [10] and Hu [11] have developed a technique using 5,5′-dithiobis-(2-nitrobenzoic) acid (DTNB) in other words Ellman’s reagent as a chromogen to measure the thiol levels.

Recently, Erel and Neselioglu [12] modified Ellman’s method. In this new method, it was aimed both thiol and disulfide levels in blood. Firstly, available thiol in serum/plasma was detected without pretreatment. This first result was accepted as native thiol (NT). Then, a pretreatment step was carried out to reduce dynamic disulfide bonds to free sulfhydryl groups by using sodium borohydrate (NaBH4). Second measurement was performed and second result was accepted as total thiol (TT). In this step, extra reduction of DTNB and further reduction of formed disulfide bonds may occur. To prevent this positive interference, remaining NaBH4 remnants were completely removed using formaldehyde. Lastly, half of the difference between TT and NT was accepted disulfide. During measurement procedure, 2-mercaptoethanol was used to achieve a linear calibration curve. Also, they used two wavelengths, main wavelength 415 nm, secondary wavelength 700 nm (optionally bichromatic). In this end-point assay, the first absorbance was taken before the mixing of Reagent 2 and Reagent 3 and the last absorbance was taken when the reaction trace draws a plateau. Reagent ingredients and volumes were presented in Table. In this method, thiol (-SH) was represented with NT, while oxidized thiol (-S-S) was represented by disulfide. Total thiol is composed of sum of the NT and disulfide levels. This method have analyzed NT, total thiol (TT), disulfide levels and ratios of disulfide to native thiol, disulfide to total thiol and native to total thiol to elucidate dynamic TDH in organism.

**Table:** Thiol disulfide measurement procedure according to Erel and Neselioglu [12].

Insert	Volume (μL)	Ingredient
Reagent 1*	10	10 mM NaCl in methanol–water solution, 50 v/v
Reagent 1**	10	10 mM NaBH4 in methanol–water solution, 50 v/v
Reagent 2	110	6.715 mM formaldehyde and 10.0 mM EDTA in tris buffer, 100 mM, pH: 8.2
Reagent 3	10	10 mM DTNB in methanol
Sample	10	Serum or plasma

*for native thiol, **for total thiol.

Beside to the colorimetric methods, several measurement methods were developed such as fluorometric methods [13], bioluminescence analysis [14], chromatographic methods [15].

### 2.2. Researches on thiol-disulfide homeostasis 

During last years, TDH has started to be measured more widely. Therefore, TDH has started to be understood more exactly. Dysregulated TDH has been implicated several disorders with unknown etiology. A growing body of evidence has demonstrated that TDH is involved in the several diseases. Researchers have established that changing in NT, TT, disulfide levels and/or ratios. These results may elucidate some pathogenic mechanism or may be a predictor indicating diagnostic clue, prognostic marker or therapeutic sign.

#### 2.2.1. Cardiac pathologies

In a study investigating association between TDH and severity of coronary atherosclerosis, high syntax score was found to be associated with decreased NT level and NT to disulfide ratio [16]. Also, it has been demonstrated that NT, TT and disulfide levels of stable angina pectoris and acute myocardial infarction patients were significantly lower than control patients [17,18]. Protecting power of extracellular cysteine pairs in disulfides protect for the reactive thiol groups and supporting effect on protein stability and function may be reason for lower disulfide level [19]. Lower disulfide levels may result from mentioned mechanism.

It has proposed that total thiol is independently diagnostic predictor of coronary syndrome X [20]. Also, decreased NT and increased disulfide levels are associated with coronary artery ectasia [21] and decreased NT to disulfide ratio was independently associated with slow coronary flow [22]. Additionally in a study investigating association between childhood obesity, reduced thiol parameters were decreased whereas oxidized thiol parameters were increased [23]. 

In both primary hypertension and masked hypertension patients, disulfide and ratios in favor of disulfide were found to be increased. Furthermore disulfide to NT ratio to be an independent indicator of both systolic and diastolic blood pressure [24–26]. 

In an interesting study, researchers have investigated whether TDH can predict the occurrence of anthracycline-induced cardiac toxicity. They were concluded that NT may elicit more objective data for physicians to detect which patients have high risk for cardiac toxicity [27]. 

#### 2.2.2. Endocrine disorders

Authors have demonstrated that NT and TT levels of prediabetes, type 1 and type 2 diabetes mellitus were lower while disulfide levels and disulfide to NT and disulfide to TT ratios were higher in patients compared to healthy volunteers [28–32].

Authors have demonstrated that TDH shifted toward disulfide formation in patients with autoimmune subclinical hypothyroidism and thyroid autoantibodies are positively correlated with disulfide to NT ratio [33]. 

#### 2.2.3. Neurological diseases

It has been established that NT ant TT levels of Alzheimer’s disease, diabetic axonal polyneuropathy patients and children with febrile seizures were found to be statistically lower than controls. On the contrary, disulfide/TT ratio was found to be statistically higher than controls [34–36].

Authors have demonstrated that in epileptic patients, TDH was no affected. This unchanged homeostasis may result in both selecting patients taking mono- or poly-antiepileptic medication and preferring seizure-free period in collection of patient samples [37]. 

It has been demonstrated increased NT and TT levels in the attention deficit hyperactivity disorder and migraine patients [38,39].

It has been demonstrated that disulfide levels and ratios of disulfide to NT and disulfide to TT were significantly higher in multiple sclerosis patients in the relapse than in patients in the remission [40]. Similarly, it has been demonstrated that NT and TT levels in Parkinson’s disease, panic disorder and acute ischemic stroke patients were detected to be significantly lower than healthy control subjects. Furthermore, it has been demonstrated that lower NT levels were associated with higher infarct volume and higher National Institutes of Health Stroke Scale (NIHSS) score [41–43].

Headache including migraine- and tension-type headache in childhood was investigated in a study. It has been established that there was no significant difference in patient group compared to control group in terms of TDH. However, it has been established that disulfide to NT and disulfide to TT ratios were higher in the migraine group. In tension-type headache group, a negative correlation was found between thiol levels and Pediatric Migraine Disability Assessment (PedMIDAS) [44].

#### 2.2.4. Psychiatric diseases

It has been demonstrated that NT, TT levels were significantly lower in schizophrenia and heroin addiction patients. Furthermore, authors have demonstrated a negative correlation between NT, TT levels and Positive and Negative Syndrome Scale (PANSS) [45,46].

Disruptive episodes of mania/hypomania and depression are called as bipolar affective disorder. In patients group, NT and TT levels were lower than remission and control group whereas no significant difference is detected between groups [47].

Repetitive transcranial magnetic stimulation (rTMS), a treatment modality based on magnetic pulses administered to cerebral cortex, may result in decreased serum NT and TT levels. It has been proposed that decreased thiol levels were associated with decreased inflammatory processes and decreased antioxidant activity [48]. 

#### 2.2.5. Respiratory system diseases

In acute pulmonary embolism and childhood asthma patients, it has been established that disulfide level and disulfide to NT ratio of patient group were found to be higher compared to control group [49,50]. Contrarily, in chronic obstructive pulmonary disease, asthma and asthma-chronic obstructive pulmonary disease overlap syndrome patients, TDH parameters were found similar among three groups. Furthermore, TDH parameters were not changed among smokers, nonsmokers and ex-smokers[51].

Authors have shown that NT, TT and disulfide levels in nonsmall cell lung cancer patients were significantly decreased compared with control subjects. It has been suggested that decrease of TDH parameters might be the prognostic marker of the tumor aggression and malignant disease [52]. 

#### 2.2.6. Head and neck pathologies

Authors have demonstrated that NT levels were significantly lower in patients with benign paroxysmal positional vertigo, seasonal allergic rhinitis, Bell’s palsy, obstructive sleep apne and nasal polyposis [53–57]. 

#### 2.2.7. Gastrointestinal system disorders

It has been demonstrated that in patients with celiac disease, acute pancreatitis and inflammatory bowel diseases NT and TT levels were found to be lower, whereas disulfide level was found to be higher compared to healthy subjects [58–60]. 

#### 2.2.8. Infectious diseases

In acute brucellosis, Crimean-Congo hemorrhagic fever, viral and bacterial tonsillopharyngitis NT and TT levels were found to be lower in patients than healthy control subjects. [61-63].

#### 2.2.9. Rheumatologic diseases

Familial Mediterranean fever (FMF) is found to be associated with lower NT and TT levels than healthy control subjects. During attack periods, it has been demonstrated that NT and TT levels are found to be lower than attack free periods. In fact it has been established that NT and TT levels were associated with colchicine dosage [64]. Additionally, authors have investigated FMF in terms of effect of different mutations in the MEFV gene on TDH. Authors have proposed that TDH was not sufficient indicator in different mutations in MEFV gene. Nevertheless, it has been demonstrated lower NT, TT and disulfide levels, disulfide to NT and disulfide to TT ratios in FMF patients. Authors have explained these decreased levels transformation form disulfide to S-nitrosothiol, sulfenic acid, sufinic acid and sufonic acid [65].

It has been demonstrated that TT levels were significantly lower in ankylosing spondylitis and juvenile idiopathic arthritis patients compared to control group. Additionally, authors have proposed that thiol levels might be a useful marker of disease activation and there was a significant negative correlation TT levels and visual analog scale (VAS); TT levels and Bath Ankylosing Spondylitis Disease Activity Index (BASDAI) [66, 67]. Additionally, authors have demonstrated that decreased native thiol and total thiol levels and increased WOMAC score and disulphide levels were independently associated with increased risk of late-stage osteoarthritis [68].

Fibromyalgia syndrome is related to thiol-disulfide imbalance shifted to the reductive state according to a recent paper. In this study, NT levels were found to be significantly higher and disulfide levels were found to be significantly lower in patients with fibromyalgia syndrome than control group. This situation was connected with proliferative alteration explaining with higher NT and lower disulfide levels [69].

#### 2.2.10. Dermatologic disorders

Thiol-disulfide homeostasis in patients with basal cell carcinoma, psoriasis and seborrheic dermatitis has changed as the increased NT. It has been suggested that increased NT levels cause proliferation, whereas reduced NT along with the increased disulfide results in cell apoptosis [70–72].

It has been demonstrated that TDH was shifted towards to disulfide formation in patients with chronic spontaneous urticaria whereas TDH was no changed in patients with acute urticarial [73].

In atopic dermatitis patients, serum disulfide levels and disulfide to NT and disulfide to TT ratios were found to be lower compared with healthy control children. Moreover, disulfide to NT ratio was positively correlated and NT to TT ratio was negatively correlated with higher scores assessing atopic dermatitis severity. Authors have proposed that decreased disulfide level might result from rebound phenomenon to continue protective influence by facilitating the neutralization of oxidative state [74].

It has been demonstrated that TDH was no changed in patients with alopecia areata, an autoimmune disease characterized scar-free hair loss. This may result from patients which has mild alopecia areata disease [75].

#### 2.2.11. Ophthalmic disorders

Authors have established that NT and TT levels were lower whereas disulfide level was higher in keratoconus and pseudoexfoliation syndrome patients compared with control group [76–78]. 

Native thiol and TT levels were found to be lower in central serous chorioretinopathy and macular degeneration patients compared to control subject [79–81].

In another study, authors have investigated age-related macular degeneration and they have established that NT levels were significantly decreased and disulfide levels were significantly increased in patients compared to control subjects [82]. 

#### 2.2.12. Obstetric and gynecologic disorders

As one most common endocrine disease, polycystic ovary syndrome has been shown to be related to higher thiol and lower disulfide levels. Authors have proposed that antioxidants hinder the atresia of antral follicles and the thiols play an essential role in cell proliferation, division and apoptosis. Elevated thiol levels may result from anti-oxidant response to oxidative load due to obesity or a consequence of physiological changes in polycystic ovary syndrome such as anovulation, multiple follicular development and apoptosis [83]. In another study investigating polycystic ovary syndrome, NT and TT levels were found to be significantly lower in patients with overweight polycystic ovary syndrome than normal weight polycystic ovary syndrome and oxidative stress was more marked in overweight polycystic ovary syndrome. Furthermore, it has been demonstrated that lipid accumulation index was statistically significantly higher than in patients with overweight polycystic ovary syndrome than those in other three groups. Authors have established that lipid accumulation index and TDH may play an important role in the pathogenesis of cardiovascular disease in polycystic ovary syndrome [84].

In a study investigating postmenopausal osteoporosis it has been demonstrated that disulfide to NT ratio was higher in patients after adjustment for age and menopause duration and there was a negative correlation between the disulfide to NT ratio and bone mineral density of the lumbar vertebrae [85]. 

Authors investigated whether there is an association between TDH and premature ovarian failure. It has been demonstrated that TDH was shifted in favor of disulfide formation [86]. In patients with uterine myoma it has been demonstrated that NT, TT and disulfide levels were found to be lower than healthy control subjects. These results have been commented that proliferative phase of uterine myoma might be a possible cause and disulfide levels were found to be lower than control group [87]. It has been demonstrated that NT and TT levels in patients with endometriosis were lower than control group [88].

It has been demonstrated that TDH was shifted in favor of oxidative status in preeclampsia, pregnant women diagnosed with idiopathic recurrent pregnancy loss, pregnant women diagnosed with FMF, pregnant women with fetal neural tube defects and vaginitis patients [89–93].

There are several studies investigating association between TDH and gestational diabetes mellitus [94–97]. It has been demonstrated that NT and TT levels were found to be lower than control subjects and decreased NT levels have an increased risk of possible adverse perinatal outcomes [95]. It has been demonstrated that thiol levels were significantly lower whereas disulfide levels were significantly higher in patients with gestational diabetes mellitus compared with both pregnant women with impaired glucose tolerance and uncomplicated pregnant subjects [96]. Authors have investigated pregnant women with gestational diabetes mellitus, obese pregnant women and healthy pregnant women in terms of TDH of cord blood. Native thiol levels were found to be lower in patients with gestational diabetes mellitus compared to other two groups. Total thiol levels were found to be lower in patients with gestational diabetes mellitus compared to obese pregnant women. Disulfide levels were found to be higher in patients with gestational diabetes mellitus compared to healthy pregnant women and in obese pregnant compared to healthy pregnant women. Authors have suggested that infants of obese or diabetic mothers are exposed to increased oxidative stress [94]. In another study, authors have demonstrated that 50 grams glucose challenge test-positive pregnant women have increased disulfide levels after glucose loading compared to baseline [97].

Thiol-disulfide homeostasis has been investigated in patients with abortus imminens. It has been demonstrated that NT and TT levels were found to be lower than control group. Authors have proposed that during pregnancy, insufficient antioxidant defense may be an etiologic factor in the pathogenesis of abortus imminens [98].

It has been demonstrated that NT ad TT levels were significantly lower and disulfide level was significantly higher in pregnant women with hyperemesis gravidarum and intrahepatic cholestasis and in fetus with nuchal cord during labor than control group [99–101]. 

In another study, authors have demonstrated that maternal NT to TT ratio, NT and TT levels were significantly lower while ratios of disulfide to NT and disulfide to TT were significantly higher in pregnancies complicated by intra uterine growth restriction compared to control subjects. Authors have proposed that decreased NT and TT levels might diminish maternal serum H2S level and inhibit its vasodilatation effect through nitric oxide synthesis [102].

It has been demonstrated that NT and TT levels were lower and disulfide to NT and disulfide to TT ratios were higher in pregnancies complicated by obstructive sleep apnea syndrome compared to nonpregnant control subjects [103]. 

At birth, the timing of umbilical cord clamping has important for cardiovascular system, pulmonary system and blood volume of neonate. In a study investigating effect of three different cord clamping procedures (i.e. early clamping, delayed clamping, and cord milking) on TDH, it has been demonstrated that NT and TT levels were statistically significantly lower in the early clamping group compared with the delayed clamping and cord milking group as an indicator of oxidative stress. In conclusion, they have recommended delayed cord clamping and cord milking due to beneficial effects on the neonates [104]. 

In a study investigating TDH in preterm infant, researchers have collect patient blood at baseline, first week and third week after birth. It has been demonstrated that NT and TT levels were increased in each analysis and disulfide levels and disulfide to NT and disulfide to TT ratios were increased at first week and decreased at third week. The ratio of NT to TT was decreased at first week and increased at third week. Authors have proposed that increased thiol levels might result from cysteine containing amino acid administration or breast milk that enhances antioxidative defense compared to enteral feeding. Moreover, increased disulfide level at first week may due to oxidative damaged-treatment including phototherapy and antibiotics. Furthermore, it is speculated that healing from respiratory diseases might contribute to diminish in disulfide levels at third week [105]. 

#### 2.2.13. Urological disorders

In a study executed on prostate cancer patients, authors have compared prostate cancer patients before and six month after radical prostatectomy operation and control group in term of TDH. In the study, NT, TT and disulfide levels were found to be lower in patients before operation compared to control subjects. There was a significant negative correlation between levels of prostate specific antigen and levels of NT and TT in patient group before operation and control group. Similarly, NT, TT and disulfide levels were found to be lower in patients after operation compared to control subjects. Furthermore, authors have stated that TDH begins to shift towards thiols following operation [106]. Similarly, authors have demonstrated that NT and TT levels decreased in patients underwent transrectal ultrasound guided prostate biopsy [107].

It has been demonstrated that TDH has shifted towards disulfide in patients with varicocele as one of the most common causes of male infertility [108].

#### 2.2.14. Occupational diseases

In two study investigating TDH in operating theater personnel and asphalt workers, authors have demonstrated that TDH has shifted to disulfide formation resulting from increased disulfide level, increased disulfide to NT ratio [109, 110]. 

In a study investigating occupationally arsenic-exposed workers in terms of TDH, disulfide level, disulfide to NT ratio and disulfide to TT ratio were found to be increased in workers exposed to arsenic, lead and asphalt fume than control group. Furthermore, it has been demonstrated that there are positive correlations between urinary arsenic level and disulfide level and urinary arsenic level and disulfide to NT ratio. Also, a positive correlation was detected between lead and disulfide levels [111–113]. 

It has been demonstrated that NT levels were found to be significantly lower in professionals working in radiation environments than control subjects. However, there was no difference between two groups in terms of TT and disulfide levels. The results have been explained that NT would only have been affected by initial stages of these processes [114].

When TDH parameters were investigated silica and trichloroethylene exposure group, it has been established higher disulfide levels in exposed workers than healthy volunteers. Furthermore, there were a negative correlation between NT, TT, NT to TT ratio and urinary trichloroethylene levels and a positive correlation between disulfide to NT and disulfide to TT ratios and urinary trichloroethylene levels [115,116]. 

#### 2.2.15. Other disorders

In an interesting study, authors have investigated TDH parameters in gunshot injuries. It has been demonstrated that NT, TT and disulfide levels were decreased whereas disulfide to NT and disulfide to TT ratios were increased in patient with gunshot injury. Furthermore, NT levels were found to be an independent indicator of Revised Trauma Scale (RTS) and Glasgow Coma Scale (GCS). Authors have speculated that low NT levels might result from attempt to detoxify oxidative stress and ROS generated due to trauma [117].

The authors have demonstrated that NT and TT levels are found to be lower and disulfide level was found to be higher in patients with acute appendicitis compared to control group. In fact this shift towards disulfide formation was valid in perforated appendicitis patients compared to nonperforated appendicitis patients [118]. Similarly, in breast cancer patients, NT and TT levels are found to be lower and disulfide level was found to be higher than control subjects [119].

In a study investigating effect of elective laparoscopic cholecystectomy and open inguinal-femoral hernia repair operation on TDH, the authors have demonstrated that during operation, NT, TT and disulfide levels were found to be decreased. Additionally, 24 h after laparoscopic surgery, NT, TT and disulfide levels were found to be increased but not return to preoperative levels [120]. 

In operated aortic aneurysm and acute aortic syndrome patients, NT and TT levels were increased and disulfide level and disulfide to NT ratio were decreased after 6 months of operation. Authors have concluded that thiol and disulfide levels are distinctive tests between healthy subjects and aneurysm or acute aortic syndrome [121].

It has been demonstrated that in patients with carbon monoxide poisoning and smokers NT and TT levels were decrease whereas disulfide level was increased [122, 123].

Authors have demonstrated that NT, TT and disulfide levels decreased in patient with multiple myeloma, a malignancy of bone morrow. Because the ratios were similar in all groups, authors have asserted that there is a balance between thiol and disulfide levels and the balance determine the systemic effects [124].

It has been demonstrated that TDH parameters were no changed in maple syrup urine disease patients. Authors have decided that good metabolic control and proper dietary compliance in maple syrup urine disease patients may prevent oxidative stress [125]. 

Recently, it has been provided that thiol-disulfide homeostasis might be a notable key for evaluating the severity of burns and predicting the survival [126].

## 3. Conclusion

Thiol-disulfide homeostasis is reversal of thiol oxidation in proteins and represents the levels of thiols and disulfides. Thiol-disulfide homeostasis is an important parameter associated with several biochemical processes. Dysregulated thiol-disulfide homeostasis has been implicated several disorders with unknown etiology. The evidence so far has proposed that thiol-disulfide homeostasis is an important issue and needs to be elucidated wholly.

## References

[ref1] (2016). Thiol-disulfide exchange reactions in the mammalian extracellular environment. Annual Review of Chemical Biomolecular Engineering.

[ref2] (1984). Lipid peroxidation, oxygen radicals, cell damage, and antioxidant therapy. Lancet.

[ref3] (2007). ROS as signalling molecules: mechanisms that generate specificity in ROS homeostasis. Nature Reviews Molecular Cell Biology.

[ref4] (2004). Cysteine/cystine couple is a newly recognized node in the circuitry for biologic redox signaling and control. The FASEB Journal.

[ref5] (2002). Redox analysis of human plasma allows separation of pro-oxidant events of aging from decline in antioxidant defenses. Free Radical Biology Medicine.

[ref6] (2018). How are proteins reduced in the endoplasmic reticulum?. Trends in Biochemical Sciences.

[ref7] (2008). Determination of thiols and disulfides via HPLC quantification of 5-thio-2-nitrobenzoic acid. Journal of Pharmaceutical and Biomedical Analysis.

[ref8] (2014). Disulfide-containing parenteral delivery systems and their redox-biological fate. Journal of Controlled Release.

[ref9] (2019). Oxidative stress indexes for diagnosis of health or disease in humans. Oxidative Medicine and Cellular Longevity.

[ref10] (1959). Tissue sulfhydryl groups. Archives of Biochemistry Biophysics.

[ref11] (1994). Measurement of protein thiol groups and glutathione in plasma. Methods Enzymol.

[ref12] (2014). A novel and automated assay for thiol/disulphide homeostasis. Clinical Biochemistry.

[ref13] (1966). A fluorometric assay for glutathione. Analytical Biochemistry.

[ref14] (1984). Micelle-enhanced chemiluminescence and application to the determination of biological reductants using lucigenin. Analytical Chemistry.

[ref15] (1980). Estimation of glutathione in rat liver by reversed-phase high-performance liquid chromatography: separation from cysteine and gamma-glutamylcysteine. Journal of Chromatography.

[ref16] (2015). Association of thiol/disulfide ratio with syntax score in patients with NSTEMI. Scandinavian Cardiovascular Journal.

[ref17] (2016). The relation of serum thiol levels and thiol/disulphide homeostasis with the severity of coronary artery disease. Kardiologia Polska.

[ref18] (2015). A novel oxidative stress marker in acute myocardial infarction; thiol/disulphide homeostasis. The American Journal of Emergency Medicine.

[ref19] (2012). Disulfide bonding in protein biophysics. Annual Review of Biophysics.

[ref20] (2016). Evaluation of thiol levels, thiol/disulfide homeostasis and their relation with inflammation in cardiac syndrome X. Coronary Artery Disease.

[ref21] (2016). Plasma thiols and thiol-disulfide homeostasis in patients with isolated coronary artery ectasia. Atherosclerosis.

[ref22] (2016). Association of thiol disulfide homeostasis with slow coronary flow. Scandinavian Cardiovascular Journal.

[ref23] (2017). Dynamic thiol/disulphide homeostasis as a novel indicator of oxidative stress in obese children and its relationship with inflammatory-cardiovascular markers. The Anatolian Journal of Cardiology.

[ref24] (2018). Evaluation of the level of dynamic thiol/disulphide homeostasis in adolescent patients with newly diagnosed primary hypertension. Pediatric Nephrology.

[ref25] (2016). Is disulphide/thiol ratio related to blood pressure in masked hypertension? Clinical and Experimental Hypertension.

[ref26] (2016). Dynamic thiol/disulphide homeostasis in patients with newly diagnosed primary hypertension. Journal of American Society of Hypertension.

[ref27] (2017). The Role of Thiol/Disulphide Homeostasis in Anthracycline Associated Cardiac Toxicity. International Heart Journal.

[ref28] (2016). Determination of thiol/disulphide homeostasis in type 1 diabetes mellitus and the factors associated with thiol oxidation. Endocrine.

[ref29] (2018). The significance of thiol/disulfide homeostasis and ischemia-modified albumin levels to assess the oxidative stress in patients with different stages of diabetes mellitus. Scandinavian Journal of Clinical Laboratory Investigation.

[ref30] (2020). The variation of disulfides in the progression of type 2 diabetes mellitus. Experimental and Clinical Endocrinology & Diabetes.

[ref31] (2015). How does thiol/disulfide homeostasis change in prediabetic patients. Diabetes Research and Clinical Practice.

[ref32] (2019). How does thiol/disulfide homeostasis change in children with type 1 diabetes mellitus. Diabetes Research and Clinical Practice.

[ref33] (2016). Dynamic thiol/disulfide homeostasis in patients with autoimmune subclinical hypothyroidism. Endocrine Research.

[ref34] (2016). A novel oxidative stress marker in patients with Alzheimer’s disease: dynamic thiol-disulphide homeostasis. Acta Neuropsychiatr.

[ref35] (2017). /disulfide homeostasis as a novel indicator of oxidative stress in children with simple febrile seizures. Neurological Sciences.

[ref36] (2018). Impaired thiol-disulphide homeostasis in patients with axonal polyneuropathy. Neurological Research.

[ref37] (2016). Evaluation of Dynamic Thiol-Disulphide Homeostasis in Patients with Epilepsy. Epilepsi: Journal of the Turkish Epilepsi Society.

[ref38] (2017). Dynamic thiol/disulfide homeostasis in children with attention deficit hyperactivity disorder and its relation with disease subtypes. Comprehensive Psychiatry.

[ref39] (2016). A novel oxidative stress marker in migraine patients: dynamic thiol-disulphide homeostasis. Neurological Sciences.

[ref40] (2016). Dynamic thiol-disulphide homeostasis in patients with multiple sclerosis. World Journal of Neuroscience.

[ref41] (2017). Impairment of dynamic thiol-disulphide homeostasis in patients with idiopathic Parkinson’s disease and its relationship with clinical stage of disease. Clinical Neurology and Neurosurgery.

[ref42] (2017). Thiol-disulfide homeostasis in patients with panic disorder. International Journal of Clinical Medicine.

[ref43] (2016). Dynamic thiol-disulfide homeostasis in acute ischemic stroke patients. Acta Neurologica Belgica.

[ref44] (2017). Headache in children and dynamic thiol/disulfide balance evaluation with a new method. Neurological Sciences.

[ref45] (2017). Thiol/disulfide homeostasis in untreated schizophrenia patients. Psychiatry Research.

[ref46] (2017). Thiol/disulphide homeostasis in men with heroin addiction. Düşünen Adam Dergisi.

[ref47] (2018). Thiol/disulphide homeostasis in bipolar disorder. Psychiatry Research.

[ref48] (2017). The impact of repetitive transcranial magnetic stimulation on oxidative stress in subjects with medication-resistant depression. The Journal of ECT.

[ref49] (2016). Association of thiol disulfide homeostasis with childhood asthma. Journal of Pediatric Biochemistry.

[ref50] (2017). Are the thiol/disulfide redox status and HDL cholesterol levels associated with pulmonary embolism? Thiol/disulfide redox status in pulmonary embolism. Clinical Biochemistry.

[ref51] (2016). Comparison of thiol/disulphide homeostasis parameters in patients with COPD, asthma and ACOS. European Review for Medical and Pharmacological Sciences.

[ref52] (2016). Thiol/disulfide homeostasis: a prognostic biomarker for patients with advanced non-small cell lung cancer?. Redox Report.

[ref53] (2018). Oxidative status in patients with benign paroxysmal positional vertigo. The Journal of International Advanced Otology.

[ref54] (2016). A new oxidative stress marker for thiol-disulphide homeostasis in seasonal allergic rhinitis. American Journal of Rhinology & Allergy.

[ref55] (2017). Thiol/disulphide homeostasis in Bell’s palsy as a novel pathogenetic marker. Clinical Otolaryngology.

[ref56] (2017). Thiol/disulfide homeostasis as a novel indicator of oxidative stress in obstructive sleep apnea patients. Laryngoscope.

[ref57] (2016). A novel method for determining the relation between nasal polyposis and oxidative stress: the thiol/disulphide homeostasis. Acta Oto-Laryngololica.

[ref58] (2017). Thiol/disulphide homeostasis in celiac disease. World Journal of Gastrointestinal Pharmacology and Therapeutics.

[ref59] (2016). The dynamic thiol/disulphide homeostasis in inflammatory bowel disease and its relation with disease activity and pathogenesis. International Journal of Colorectal Disease.

[ref60] (2018). Dynamic thiol/disulphide homeostasis in acute pancreatitis. Turkish Journal of Gastroenterology.

[ref61] (2017). A retrospective controlled study of thiol disulfide homeostasis as a novel marker in Crimean Congo hemorrhagic fever. Redox Report.

[ref62] (2017). Impaired Thiol-Disulfide Balance in Acute Brucellosis. Japanese Journal of Infectious Diseases.

[ref63] (2017). Alteration of thiol-disulphide homeostasis in acute tonsillopharyngitis. Redox Report.

[ref64] (2017). the thiol/disulfide imbalance be a predictor of colchicine resistance in familial Mediterranean fever. Journal of Korean Medical Science.

[ref65] (2018). Does thiol-disulphide balance show oxidative stress in different MEFV mutations?. Rheumatology International.

[ref66] (2016). Thiol/disulfide homeostasis in patients with ankylosing spondylitis. Bosnian Journal of Basic Medical Sciences.

[ref67] (2018). Changes in thiol/disulfide homeostasis in juvenile idiopathic arthritis. Pediatrics International.

[ref68] (2019). Is there a relationship between dynamic thiol/disulfide homeostasis and osteoarthritis progression?. Archives of Physiology and Biochemistry.

[ref69] (2017). Dynamic thiol/disulphide homeostasis in patients with fibromyalgia. Archives of Rheumatology.

[ref70] (2017). Dynamic thiol/disulphide homeostasis in patients with basal cell carcinoma. Cutaneous and Ocular Toxicology.

[ref71] (2017). Dynamic thiol/disulfide homeostasis and effects of smoking on homeostasis parameters in patients with psoriasis. Cutaneous and Ocular Toxicology.

[ref72] (2020). Dynamic thiol/disulfide balance in patients with seborrheic dermatitis: a case–control study. Saudi Journal of Medicine and Medical Sciences.

[ref73] (2017). Investigation of thiol-disulphide balance in patients with acute urticaria and chronic spontaneous urticaria. Cutaneous and Ocular Toxicology.

[ref74] (2018). Association of oxidative stress and dynamic thiol-disulphide homeostasis with atopic dermatitis severity and chronicity in children: a prospective study. Clinical and Experimental Dermatology.

[ref75] (2017). Investigation of dynamic thiol-disulfide homeostasis in alopecia areata patients. Journal of Advances in Medicine and Medical Research.

[ref76] (2017). Investigation of dynamic thiol-disulphide homoeostasis in age-related cataract patients with a novel and automated assay. International Ophthalmology.

[ref77] (2017). Novel assay assessment of oxidative stress biomarkers in patients with keratoconus: thiol-disulfide homeostasis. Current Eye Research.

[ref78] (2017). Thiol disulfide homeostasis in pseudoexfoliation syndrome. Current Eye Research.

[ref79] (2016). Thiol/disulfide homeostasis in patients with central serous chorioretinopathy. Current Eye Research.

[ref80] (2018). A novel marker in acute central serous chorioretinopathy: thiol/disulfide homeostasis. International Ophthalmology.

[ref81] (2016). A novel tool for the assessment oxidative stress in age-related macular degeneration: thiol/disulfide homeostasis revisited. Current Eye Research.

[ref82] (2017). Dynamic thiol/disulfide homeostasis in patients with age-related macular degeneration. Arquivos Brasileiros de Oftalmologia.

[ref83] (2017). Dynamic thiol-disulphide status in polycystic ovary syndrome and its association with the pathogenesis of the disease. Gynecologic and Obstetric Investigation.

[ref84] (2016). The association of thiol/disulphide homeostasis and lipid accumulation index with cardiovascular risk factors in overweight adolescents with polycystic ovary syndrome. Clinical Endocrinology.

[ref85] (2017). Thiol/disulfide homeostasis in postmenopausal osteoporosis. Journal of Endocrinological Investigation.

[ref86] (2017). The use of thiol/disulfide as a novel marker in premature ovarian failure. Gynecologic and Obstetric Investigation.

[ref87] (2017). Dynamic thiol/disulphide homeostasis in patients with Uterine Myoma. European Journal of Obstetric & Gynecology Reproductive Biology.

[ref88] (2016). Evaluation of oxidative stress markers and intra-extracellular antioxidant activities in patients with endometriosis. European Journal of Obstetric & Gynecology Reproductive Biology.

[ref89] (2018). Dynamic thiol/disulphide homeostasis in patients with vaginitis. Journal of Gynecology and Obstetrics.

[ref90] (2015). Serum thiol/disulphide homeostasis in preeclampsia. Hypertension in Pregnancy.

[ref91] (2016). Thiol/disulphide homeostasis in pregnant women with familial Mediterranean fever. Redox Report.

[ref92] (2016). Thiol/disulfide homeostasis in patients with idiopathic recurrent pregnancy loss assessed by a novel assay: Report of a preliminary study. Journal of Obstetrics & Gynaecology Research.

[ref93] (2019). Oxidative-Antioxidative Markers in Pregnant Women with Fetal Neural Tube Defects. Fetal and Pediatric Pathology.

[ref94] (2017). Impact of gestational diabetes mellitus and maternal obesity on cord blood dynamic thiol/disulfide homeostasis. Fetal and Pediatric Pathology.

[ref95] (2016). Thiol/disulfide homeostasis in predicting adverse perinatal outcomes at 24-28 weeks of pregnancy in gestational diabetes. The Journal of Maternal-Fetal & Neonatal Medicine.

[ref96] (2016). Altered maternal serum dynamic thiol-disulfide interchange reactions in pregnant women with gestational diabetes mellitus. Gynecology Obstetrics & Reproductive Medicine.

[ref97] (2018). The effect of the 50 g glucose challenge test on the thiol/disulfide homeostasis in pregnancy. Fetal and Pediatric Pathology.

[ref98] (2017). Investigation of serum thiol/disulphide homeostasis in patients with abortus imminens. The Journal of Maternal-Fetal & Neonatal Medicine.

[ref99] (2015). Dynamic thiol-disulfide homeostasis in hyperemesis gravidarum. Journal of Perinatology.

[ref100] (2017). An alternative method for measuring oxidative stress in intrahepatic cholestasis of pregnancy: thiol/disulphide homeostasis. The Journal of Maternal-Fetal & Neonatal Medicine.

[ref101] (2018). Evaluation of fetal serum thiol/disulphide homeostasis in deliveries complicated by nuchal cord. The Journal of Maternal-Fetal & Neonatal Medicine.

[ref102] (2018). The maternal serum thiol/disulfide homeostasis is impaired in pregnancies complicated by idiopathic intrauterine growth restriction. The Journal of Maternal-Fetal & Neonatal Medicine.

[ref103] (2017). Thiol/disulfide homeostasis in pregnant women with obstructive sleep apnea syndrome. The Journal of Maternal-Fetal & Neonatal Medicine.

[ref104] (2018). Is early cord clamping, delayed cord clamping or cord milking best?. The Journal of Maternal-Fetal & Neonatal Medicine.

[ref105] (2017). Evaluation of dynamic thiol-disulfide homeostasis in very low-birth-weighted preterms. The Journal of Maternal-Fetal & Neonatal Medicine.

[ref106] (2016). Dynamic thiol/disulphide homeostasis before and after radical prostatectomy in patients with prostate cancer. Free Radical Research.

[ref107] (2017). The change in serum Thiol/Disulphide homeostasis after transrectal ultrasound guided prostate biopsy. International Brazilian Journal of Urology.

[ref108] (2018). Thiol-disulphide homoeostasis as an oxidative stress marker in men with varicocele. Andrologia.

[ref109] (2017). Dynamic thiol disulphide homeostasis in operating theater personnel exposed to anesthetic gases. American Journal of Industrial Medicine.

[ref110] (2016). Thiol/disulfide homeostasis in asphalt workers. Archives of Environmental and Occupational Health.

[ref111] (2018). Alteration of thiol-disulfide homeostasis in workers occupationally exposed to arsenic. Archives of Environmental and Occupational Health.

[ref112] (2017). Dynamic disulfide/thiol homeostasis in lead exposure denoted by a novel method. Toxicology and Industrial Health.

[ref113] (2018). Occupational exposure to asphalt fume can cause oxidative DNA damage among road paving workers. American Journal of Industrial Medicine.

[ref114] (2017). Evaluation of thiol-disulphide homeostasis in radiation workers. International Journal of Radiation Biology.

[ref115] (2017). Evaluation of dynamic disulphide/thiol homeostasis in silica exposed workers. Balkan Medical Journal.

[ref116] (2016). The compromise of dynamic disulfide/thiol homeostasis as a biomarker of oxidative stress in trichloroethylene exposure. Human & Experimental Toxicology.

[ref117] (2019). Serum thiol levels and thiol/disulphide homeostasis in gunshot injuries. European Journal of Trauma and Emergency Surgery.

[ref118] (2016). A novel oxidative stress mediator in acute appendicitis: thiol/disulphide homeostasis. Mediators of Inflammation.

[ref119] (2019). Thiol-disulfide homeostasis in breast cancer patients. Journal of Cancer Research & Therapeutics.

[ref120] (2016). Changes in thiol-disulfide homeostasis of the body to surgical trauma in laparoscopic cholecystectomy patients. Journal of Laparoendoscopic & Advanced Surgical Techniques.

[ref121] (2018). Thiol/disulphide homeostasis in thoracic aortic aneurysm and acute aortic syndrome. Biomarkers in Medicine.

[ref122] (2016). Disulfide stress in carbon monoxide poisoning. Clinical Biochemistry.

[ref123] (2018). Effects of smoking on thiol/disulfide homeostasis. European Review for Medical and Pharmacological Sciences.

[ref124] (2017). Assessment of serum thiol/disulfide homeostasis in multiple myeloma patients by a new method. Redox Report.

[ref125] (2017). Evaluation of dynamic thiol/disulphide homeostasis as a novel indicator of oxidative stress in maple syrup urine disease patients under treatment. Metabolic Brain Disease.

[ref126] (2019). A remarkable point for evaluating the severity of burns: thiol-disulfide profile. Burns.

